# A new model of integrated primary-secondary care for complex diabetes in the community: study protocol for a randomised controlled trial

**DOI:** 10.1186/1745-6215-14-382

**Published:** 2013-11-12

**Authors:** Jianzhen Zhang, Letitia Burridge, Kimberley A Baxter, Maria Donald, Michele M Foster, Samantha A Hollingworth, Robert S Ware, Anthony W Russell, Claire L Jackson

**Affiliations:** 1 School of Medicine, The University of Queensland, Royal Brisbane & Women’s Hospitals, Level 8, Health Sciences Building, Building 16/910, Herston Road, Herston, QLD 4006, Australia; 2 School of Social Work and Human Services, The University of Queensland, St Lucia, QLD 4072, Australia; 3 School of Pharmacy, The University of Queensland, 20 Cornwall St, Woolloongabba, QLD 4102, Australia; 4 School of Population Health, The University of Queensland, Herston Road, Herston, QLD 4006, Australia; 5 Princess Alexandra Hospital, Ipswich Road, Woolloongabba, QLD 4102, Australia; 6 School of Medicine, The University of Queensland, St Lucia, QLD 4072, Australia

**Keywords:** Type 2 diabetes mellitus, Model of care, Randomised controlled trial, Intervention, Usual care

## Abstract

**Background:**

A new model of complex diabetes care is provided by a multidisciplinary team which incorporates general practitioner (GP) Clinical Fellows supported by an Endocrinologist and diabetes educator within a community-based general practice setting. This study evaluates the health and clinical benefits of the new model of care, assesses the acceptability of the model to patients, GPs and other health professionals, and examines the cost-effectiveness of the model.

**Methods/Design:**

The study is an open, non-inferiority randomised controlled trial with data collected at baseline, 6 and 12 months. Participants are identified from new patients on hospital-based diabetes outpatient clinic waiting lists and new GP referrals. Eligible consenting patients are randomised to either a community practice site (intervention) or a hospital site (usual care). In the intervention model, medical care is led by a GP Clinical Fellow in partnership with an Endocrinologist. Quantitative measures include clinical indicators with HbA1c as the primary outcome; patient-reported outcomes include health-related quality of life, mental health and satisfaction with care. Qualitative methods will be used to explore the perspectives and experiences of patients and providers regarding the new model of care. An economic evaluation will also be undertaken.

**Discussion:**

This model of care seeks to improve the quality and safety of healthcare at the interface between the hospital and primary care sectors for patients with complex diabetes. The study will provide empirical evidence about the impact of the model of care on health outcomes, patient and clinician satisfaction, as well as any economic impacts.

**Trial registration:**

Clinical Trials Registry Number: ACTRN12612000380897

## Background

Type 2 diabetes mellitus (T2DM) is one of the most common chronic diseases and significantly impacts healthcare systems in both the developed and developing world. Diabetes currently affects about 371 million people worldwide [1] and this figure is expected to increase to 522 million by 2030 [2]. In 2012, 11.7% of adult Australians were estimated to have diabetes. This prevalence is increasing steadily, being the fourth most frequently managed chronic disease in general practice, accounting for 6.8% of all chronic disease-related GP visits [3]. Internationally, waiting lists at specialist diabetes outpatient departments continue to grow, resulting in care that can be fragmented and inefficient [4-8]. All indications are that Australia faces similar challenges with long waiting lists and difficulties related to accessing hospital-based diabetes outpatient clinics [9-11]. It follows that diabetes is a chronic disease where the GP has a central role to play.

With appropriate support, follow-up and information technology systems, delivery of complex diabetes care in general practice can be as effective as hospital-based outpatient care [12]. The United Kingdom has introduced diabetes clinics conducted by general practitioners with special interests (GPwSIs) with demonstrated favourable improvements in HbA1c, cholesterol and blood pressure [13]. This research has demonstrated the potential of GPwSIs to take on a much greater role in diabetes screening and management to help meet the current demands on hospital outpatient services [14-17]. In addition, an evaluation of a diabetes management program based on integrated care and management between GPs and GPwSIs undertaken in Germany showed a reduction in HbA1c from baseline [18]. Similar studies in Belgium and the USA have also demonstrated improved outcomes (HbA1c, systolic blood pressure and LDL-cholesterol) for diabetes patients using shared or integrated care between GPs and specialists [19-21]. A multidisciplinary and coordinated approach to diabetes care including collaboration between practitioners can result in effective, efficient service delivery [17,22].

Recently in Australia, Russell and colleagues [23] evaluated a model of complex diabetes care using a controlled pre-post study design which incorporated the principle of a GPwSI supported by an Endocrinologist and diabetes educator within a community-based general practice setting. The study showed promising results with a trend to better glycaemic control at a reduced delivery cost for the intervention group compared to the usual care group treated at a hospital diabetes outpatient clinic. Few studies have evaluated integrated diabetes services between primary care and secondary care by increasing the capacity of primary care to meet the needs of patients who would otherwise use hospital outpatient services. Even fewer have done this using randomised controlled trials. Indeed, a recent systematic review in the area concluded that there is a need to improve the design and quality of studies investigating shared care at the primary-secondary interface [24].

The specific purpose of the work reported here is to build on the promising results of Russell and colleagues’ study by using a more rigorous study design [23]. The aim of the study is to evaluate the new model of care using a range of outcomes, the primary one being glycaemic control. Secondary outcomes include: other clinical outcomes; patient-reported outcomes, such as quality of life and mental health; the acceptability of the model to patients, GPs and other healthcare providers; and the cost-effectiveness of the model.

## Methods/Design

### Study design

The study uses a non-inferiority randomised controlled trial design. A mixed methods approach of data collection including quantitative and qualitative methods is used. Non-inferiority trials are designed to show that a novel treatment is at least no worse than a standard treatment in terms of some primary clinical endpoint [25], in this case HbA1c. Accordingly, the primary hypothesis is that glycaemic control of patients with T2DM who have their treatment delivered through management by a community-based multidisciplinary integrated primary-secondary healthcare team (including an Endocrinologist) using protocol driven care, will be comparable to glycaemic control achieved in a hospital outpatient clinic setting.

The research questions include:

1. What is the impact of the new model of care on glycaemic control and other clinical indicators compared to usual care?

2. What is the impact of the new model of care on patient-reported outcomes compared to usual care?

3. What is the cost-effectiveness and cost utility of the new model of care compared to usual care?

4. What are the perspectives and experiences of patients and health professionals regarding the new model of care?

### Study settings

There are five study sites located in the southern area of Brisbane, Australia. The intervention will be administered in three community-based complex diabetes services. Usual care will be delivered at two hospital diabetes outpatient clinics.

### Participants

Patients with complex and/or uncontrolled T2DM will be eligible to participate, and will be recruited from the two hospital diabetes outpatient clinics. Potential participants will be identified from patients who are on the current waiting list at each hospital clinic and consecutive new patients who are referred to these clinics.

#### Inclusion and exclusion criteria

Patients with T2DM will be eligible if, on receipt of referral, they are medically triaged to the most urgent assigned Category 1 or less urgent Category 2 complex diabetes patients as defined by the local health department guidelines (see Additional file 1), aged 18 years or older, referred as a new patient in the last 12 months, and living in one of three eligible catchment areas defined by the boundaries of the three community-based complex diabetes services.

Patients will be excluded if they attended one of the study hospital clinics in the previous 12 months for complex T2DM; or if they are pregnant, on haemodialysis, a renal transplant recipient, on a waiting list for transplant, terminally ill with a life expectancy <2 years, or unable to give informed consent.

### Recruitment and randomisation

Research staff located at each participating hospital will contact eligible patients by telephone to introduce the study, confirm eligibility, gain verbal consent for participation and confirm patients’ contact details. Urgent (Category 1) patients will be contacted as a priority so they can be seen within 1 to 2 weeks of referral. Eligible, interested patients will be sent an Introductory Letter, a Participant Information Sheet and a Consent Form. Patients who wish to participate will be asked to sign and return the Consent Form by post or email. Patients who refuse to participate will be treated at the hospital diabetes outpatient clinics.

After consent, each participant will be randomly allocated to the intervention or usual care group, facilitated by an online computerised centralised allocation process. Participants will be randomly assigned to the intervention or usual care group using a computer-generated random number sequence with an allocation ratio of 3:1 intervention to usual care. Randomisation will be conducted remotely and in advance, by the study statistician, who is blinded to the identity of participants. Participants will be stratified by catchment area for randomisation, and then will be randomised to either a community-based complex diabetes services site (intervention) or a hospital site (usual care). Allocation to group will be concealed to the researcher during the recruitment and consenting process. The researcher will access the secure website after the patient has provided written consent to obtain the participant’s group allocation.

**Figure 1:**
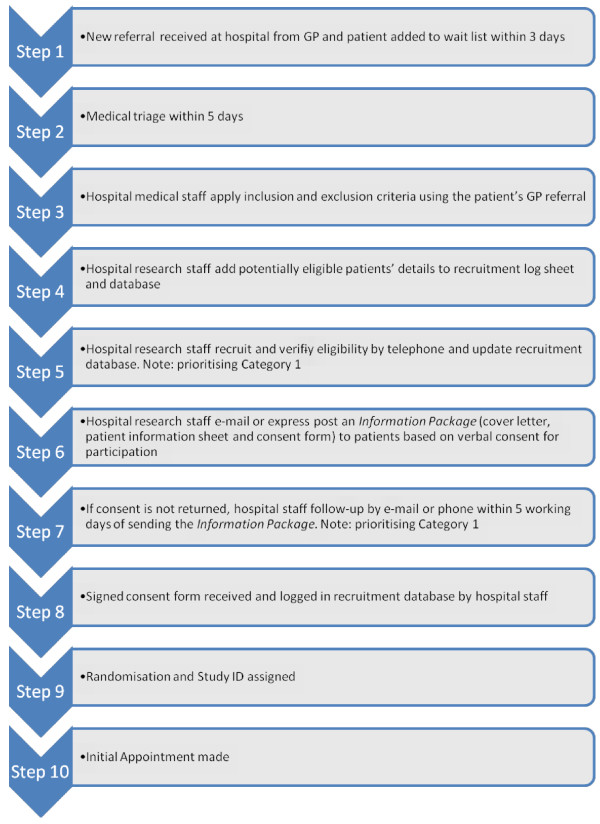
Recruitment flow chart.

Once randomised, patients will be notified of their appointment details by standard practice (that is, letter). The detailed recruitment steps are shown in Figure 1.

### Intervention

Details of the intervention model for the complex diabetes service have been reported previously [26,27]. Care in the intervention arm is provided by a multidisciplinary team consisting of an Endocrinologist, advanced-skilled GPs (known as Clinical Fellows), a credentialed diabetes educator and a podiatrist all located together in a community-based complex diabetes service. Additional allied health staff (for example, dietician, psychologist) are accessed on referral depending on patient need. The Clinical Fellows are experienced local GPs who have undertaken additional postgraduate education in advanced diabetes care, via the Master of Medicine (GP) online curriculum. This training and integrated care guidelines underpins of the model of care in the community-based clinic.

During their first visit to the complex diabetes service, patients undertake a comprehensive screening assessment by a credentialed diabetes educator (care-coordinator), following a set protocol as outlined in the diabetes management guidelines, which are evidenced-based guidelines including a detailed medical management and lifestyle management plan [27]. This includes a review of medications, diabetic history, ensuring retinal screening has been performed, foot assessment, depression screen and appropriate blood and urine testing, prior to booking for the next available medical appointment at the next available clinic. Clinics are one-half day in duration and occur every week; they involve one Endocrinologist, two to three Clinical Fellows and a diabetes educator.

During the medical appointment, patients are first assessed by one of the Clinical Fellows, who clarifies the history, examines the patient and interprets pathology results. With the patient, the Clinical Fellow drafts a management plan addressing glycaemic control, blood pressure, lipids, lifestyle, diabetes complication management and the patient’s priorities. The plan is then discussed with the attending Endocrinologist, who briefly co-consults with the patient and Clinical Fellow together to finalise the plan. This allows the Endocrinologist to reduce time spent with each patient, and to see two to three times the number of patients per clinic than possible via the traditional outpatient model. Patients initiating or altering insulin regimens are enrolled in the Insulin Stabilisation Service, where patients are contacted by phone twice weekly by the diabetes educator regarding insulin adjustment, according to defined guideline protocols. These insulin dosage adjustments are reviewed by a Clinical Fellow or Endocrinologist within the week. The patient’s GP is kept closely informed of all care management, and patients are discharged back to the care of their usual GP once glycaemic, blood pressure and lipid targets have been achieved. Patients continue to attend the clinic, but are discharged if there is no evidence of ongoing improvement following the 12-month review. The GP is advised to continue the usual cycle of care and is given some parameters for future re-referral of that individual patient.

### Usual care

Care in the usual care arm is provided in a hospital diabetes outpatient clinic. The patient’s chart is prepared and the patient then attends a ‘complication screening’ visit performed by a diabetes nurse educator (DNE) to provide education to the patient and facilitate accurate triaging of diabetes patients on the clinic waiting list. One week before the appointment, the patient is phoned to ensure they have received their appointment letter and to confirm their contact details.

Appointments are mailed to patients as they become available. The patient has an initial assessment with the Endocrinologist or clinic doctor, with correspondence regarding the management plan communicated to the GP, and further follow-up arranged at the outpatient clinic as determined by the treating Endocrinologist. An insulin stabilisation service conducted by the DNE is also offered to the patient if required.

#### Quantitative study data

**Figure 2:**
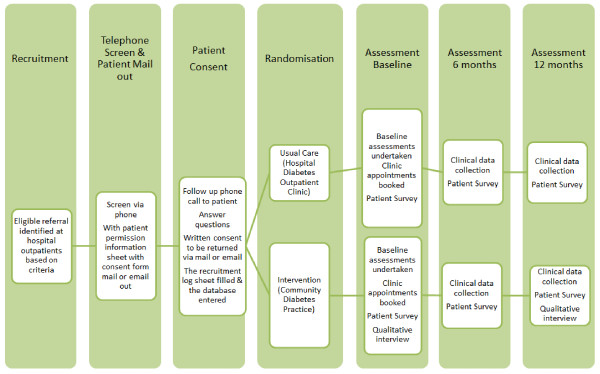
Study procedure of the T2DM Research Project.

Figure 2 displays the study procedure. Quantitative data collection will include: (1) clinical data extracted from the diabetes clinics’ databases; and (2) patient-reported outcomes collected via patient surveys. These data will be collected at baseline, 6 and 12 months. Clinical data will be extracted every 6 months from the clinics’ databases by a research project officer. General practice records will be relied on for missing clinical data. The surveys will be distributed to patients before they attend their medical appointment by the DNE at each site. All quantitative data will be stored centrally at the university research office in a purpose-designed password-protected web-based database.

#### Patient demographics

Demographic characteristics recorded include age, sex, Aboriginal or Torres Strait Islander status, language spoken at home, and length of time since diagnosis, household income, educational attainment and employment status. The data are partially collected from clinical records and partially from patient survey.

#### Clinical indicators

The primary endpoint is glycaemic control as measured by the mean change in HbA1c.

The other main clinical indicators include: blood pressure; serum cholesterol; high-density lipoprotein (HDL); low-density lipoprotein (LDL); triglycerides; serum creatinine; and estimated glomerular filtration rate (eGFR). Medications, diabetes complications, smoking status and body mass index will be recorded. In addition, health services use information, date of discharge from clinic if applicable and allied health referrals will be collected.

#### Patient-reported outcomes

We will measure patients’ quality of life and self-management, determine the levels of anxiety and depression that a patient is experiencing, and measure how satisfied patients are with the service that they receive. The following standardised scales are used:

(1) The Australian version of the SF-12v2® Health Survey is a shorter version of the SF-36v2® Health Survey (The Short Form (36) Health Survey is a survey of patient health) that uses only 12 items to measure functional health and wellbeing from the patient’s point of view [28].

(2) The Diabetes Quality of Life Scale (DQoL-brief) is a 15-item Brief Clinical Inventory which provides a total health-related quality of life score that predicts self-reported diabetes care behaviours and satisfaction with diabetes control as effectively as the full version of the instrument. In addition, it provides a vehicle for quickly screening patients for readiness and specific treatment-related concerns. It can be used to identify quality of life issues that might not arise during the typical patient-provider encounter [29].

(3) The Hospital Anxiety and Depression Scale (HADS) is a 14-item scale that generates ordinal data. Seven of the items relate to anxiety and seven relate to depression. This measure was created specifically to avoid reliance on aspects of these conditions that are also common somatic symptoms of illness (for example, fatigue and insomnia or hypersomnia). The tool is designed to detect anxiety and depression in people with physical health problems [30].

(4) The Client Satisfaction Questionnaire (CSQ-8) is used to measure patient general satisfaction across health services utilisation. It includes eight items with good psychometrics, high client and staff acceptability, and sensitivity to different levels of program quality [31]. The CSQ-8 will only be included in the survey at 6 and 12 months.

(5) The Self-management Support Scale [32] is an important component of improving chronic care delivery. It has five items and will only be used at the 6- and 12-month data collection points.

(6) Self-reported frequency of hypoglycaemic events and diabetes complications will also be measured in the patient survey.

#### Economic indicators

Direct and indirect costs for each patient will be measured using a four-item access to care scale in the patient survey [16]. This includes some non-medical direct costs (such as parking and transport) and indirect costs (such as waiting time and time lost to paid work for both patient and/or carer). Direct medical costs will include resource use and service costs, comprising staff costs (for example, consultant, diabetes educator/DNE and so on), other service costs for allied health, infrastructure costs (overheads including rental, utilities, IT support) and attendance information. The economic evaluation will comprise a cost-effectiveness analysis and a cost-utility analysis. The costs for the patient will be measured over the 12-month period of the study.

### Qualitative study data

The qualitative component incorporates: (1) semi-structured interviews with patients; (2) focus groups with health providers; and (3) semi-structured interviews with key organisational stakeholders. Qualitative data will be collected within the first 3 months and at 12 months. The qualitative data sample, aims and data collection methods are presented in Table 1. Approximately 30 to 40 intervention group patients will be purposively selected across the study sites, based on key dimensions to ensure information-rich cases that are typical of patients with T2DM. In this case, sampling will be based on two key dimensions: level of HbA1c and age of onset of diabetes, and aim to include a balance of male and female participants. Beyond these main criteria the intention is to sample for diversity based on other factors (for example, socioeconomic status, health insurance status). Participants will be excluded as potential interviewees if too unwell to manage a 30- to 45-minute interview, if hospitalised or if unable to undertake an interview for other reasons. All potential interviewees will be contacted by phone to confirm that they are willing to participate and are available for interview. If interested, a mutually suitable date and time will be arranged and a copy of the interview guide will be offered to enable the interviewee to prepare. At each community site, all health professionals involved in the management of patients with T2DM under the new model of care will be invited to participate in a site-specific focus group, including all GP Clinical Fellows and specialists, allied health and nursing personnel. At each intervention site, one key organisational stakeholder with a management and/or strategic level responsibility within the practice will be identified and invited to participate in a semi-structured interview.

**Table 1:** Summary of qualitative data collection

**Sample**	**Aim**	**Data collection**
Purposive sample of intervention group participants	Explore experiences of current care and expectations and perceptions of intervention model	Interview guided by open-ended questions within first 3 months, and at 12 months
Site-specific groups of multidisciplinary health professionals	Explore implementation of model of care and contextual factors impacting delivery and sustainability	Focus groups guided by open-ended questions within first 3 months, and at 12 months
Site-specific key stakeholders at organisational and strategic levels	Explore implementation of model of care and contextual factors impacting delivery and sustainability	Interview guided by open-ended questions within first 3 months, and at 12 months

An interview guide of open-ended questions will be used to explore patients’ expectations, perceptions and experiences of receiving the intervention model of care. Likewise, open-ended questions will also be used to explore health professionals’ and key stakeholders’ experiences of implementing the model of care and their perspectives on its benefits and sustainability.

### Sample size and power calculation

Sample size is based on the primary endpoint of HbA1c using an alpha = 0.05 and power = 80%. Based on findings from the pilot study, the sample size calculation used a pooled standard deviation in HbA1c of 1.7%. The non-inferiority margin is -0.4% and the true difference between the groups in HbA1c at 12 months is predicted to be 0.1% [26]. A randomisation ratio of 3:1 (intervention to usual) has been chosen so that the clinics at the intervention sites maintain reasonable and sustainable capacity [16]. Based on the above assumptions and adding a level of attrition of 20% between baseline and 12-month follow-up in both groups, the total sample size is estimated at 456.

### Statistical analysis plan

Baseline demographic, psychosocial and clinical characteristics of participants will be summarised and compared for differences between the intervention and usual care groups. Characteristics of participants who complete and those who withdraw will be compared.

The analysis of the primary outcome (HbA1c) will be assessed using linear regression; secondary outcomes measures will be assessed using either linear regression (continuous outcomes) or logistic regression (binary outcomes). To adjust for potential between-group imbalance, the baseline score of the outcome of interest will be included as a covariate in models. Site allocation will also be included in models as a covariate.

All analyses will be conducted on an intention-to-treat basis. Statistical significance will be based on two-tailed tests, with *P* <0.05 considered significant.

### Qualitative data analysis

All interviews and focus groups will be conducted by experienced qualitative researchers and audio-taped for transcription. The transcripts will be analysed thematically [33] to identify themes and patterns in order to understand and illustrate the expectations, perceptions and experiences of patients and health professionals who use the intervention model of care. Deviant and negative cases will be included and all themes will be verified through constant comparison and team discussion.

### Ethics

The study has been granted ethical approval by the Human Research Ethics Committee of Metro South Health Service District, Centres for Health Research of Princess Alexandra Hospital and the Medical Research Ethics Committee at The University of Queensland.

## Discussion

We present a protocol for the study design and methods to evaluate a new model of diabetes care in the community through multidisciplinary collaboration and integration across the primary-secondary interface. The model of care aims to improve the quality and safety of healthcare at the interface between hospital- and community-based primary care for patients with complex diabetes. This model has been pilot-tested in a community chronic disease management service, and the results indicated benefits for diabetes patients in comparison with usual care at the hospital diabetes outpatient clinic [23,26]. The current study builds on the promising results of Russell and colleagues’ study [23] in a larger, more robust research design and addresses the methodological issues identified in their study which utilised a non-randomised convenience sampling method. Non-inferiority randomised controlled trials are usually conducted on the premise that there is some other advantage of the treatment such as a reduction in waiting list times, reduced costs or improved access [34].

The study is important because it will provide empirical evidence about the impact on health outcomes, patient and clinician satisfaction, and economic outcomes of the improved integration of services. The results will also help us to understand the perspectives and experiences of both patients and health providers regarding the new model of care. Using a mixed methods approach will enhance the quality of the findings by enabling us to examine the model from different perspectives. It will also provide insights regarding contextual factors affecting the acceptability of the model and how it may be implemented in the future. In addition, selection bias is avoided by randomising at the level of individual patients.

However, some limitations of the study need to be considered. Participant retention in this longitudinal study will be sensitive to patient medical conditions, patient motivation as well as patient relocation out of the study area. There may be some patient issues around willingness to participate if randomly allocated to a clinic setting that is difficult to attend due to distance travel cost, and so on. Additionally, self-reported data on service use may be under-reported [35,36]. Nevertheless, every effort has been made to present and structure the patient information sheet and survey for this present study in a simple and straightforward format. Furthermore we have conducted pre-testing and pilot studies to refine the survey in an attempt to minimise item non-response and facilitate a good overall response rate [37]. In addition, efforts will be also made through rigorous implementation of recruitment and survey strategies during the data collection process, and using a best-estimation technique for item non-response to reduce missing items. Also, we cannot rule out the possibility that change in the outcomes could in part be accounted for by an observer effect whereby participants modify their behaviour, in particular their self-care and self-management regimes, in response to being involved in the trial (for example, the intensity of the study assessment) rather than in response to being involved in the model of care. To minimise this effect, we have included both a control and intervention group in the study design that undertake the same measures. Patient surveys are incorporated into the process of patient care, and are distributed to patients by Registered Nurses at the clinical study sites for patients to self-complete. Ideally, if the model of care is shown to be effective and is subsequently embedded in usual clinical practice, routinely collected clinical data could be used to reassess patient outcomes in the longer term. Finally, a longer follow-up period would further elucidate the potential to investigate the sustainability of any observed effects. The results will need to be interpreted with consideration of the inherent weaknesses of non-inferiority trials [25].

There are some challenges around planning and setting up such a complex randomised controlled trial within a health services delivery setting, such as governance issues, the building of relationships with key research stakeholders, and adjustments to planned research delivery to accommodate healthcare reform changes in hospital and community care. Nevertheless, better integration of primary and secondary care and ‘ending the blame game’ between Commonwealth and State-funded healthcare has been a key element of Australian health reform since 2007. Better integrated care leading to improved quality outcomes for communities has been underlined repeatedly by the National Health and Hospitals Reform Commission, the Council of Australian Government’s Reform Council, the National Primary Health Care Strategy, the National E-Health Transition Authority and Health Workforce Australia [38]. To date, there have been few rigorous international publications to guide success in the area. Our randomised non-inferiority trial, based around a successful cohort pilot, will progress international learning in the area of chronic disease. If effective, the intervention could be applied to other chronic conditions and extended nationwide for integrated care delivery in the future.

## Trial status

Recruitment for the trial began in December 2012. To date, we have four study sites engaged and 111 patients recruited (Control: Intervention = 28:83).

## Abbreviations

CSQ: The client satisfaction questionnaire; DNE: Diabetes nurse educator; DQoL: The Diabetes Quality of Life; GP: General practitioner; GPwSIs: General Practitioners with Special Interests; HADS: The Hospital Anxiety and Depression Scale; SF-12 Health Survey: The Short Form (12) Health Survey; T2DM: Type 2 Diabetes Mellitus.

## Competing interests

There are no competing interests.

## Authors’ contributions

JZ, LB, KB, MD, MF and SH were involved in the study design and drafted the manuscript. RW were involved in the study design, statistical analysis method and drafted the manuscript. CJ and AR led the overall study design and contributed to development of the manuscript. All authors have read and approved the final manuscript.

## Supplementary Material

Additional file 1: Triage categories for diabetes services referrals. Click here for file
