# Supplementary figures and images for: A new model of integrated primary-secondary care for complex diabetes in the community: study protocol for a randomised controlled trial
Source: Trials. 2013 Nov 12;14:382. doi: 10.1186/1745-6215-14-382 (PMC3831821; doi:10.1186/1745-6215-14-382)

**Appendix A Triage Categories for Diabetes Services Referrals**


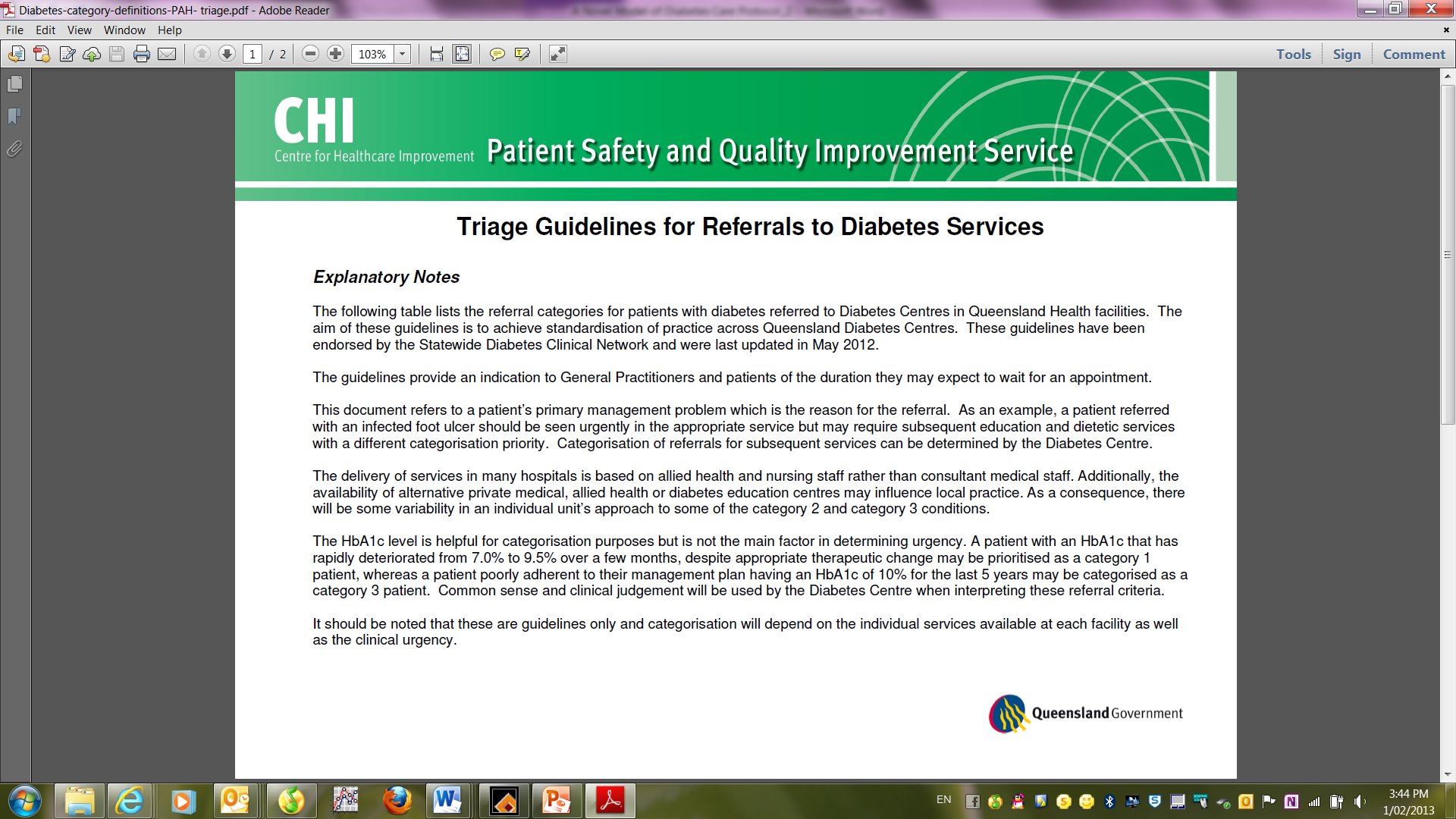

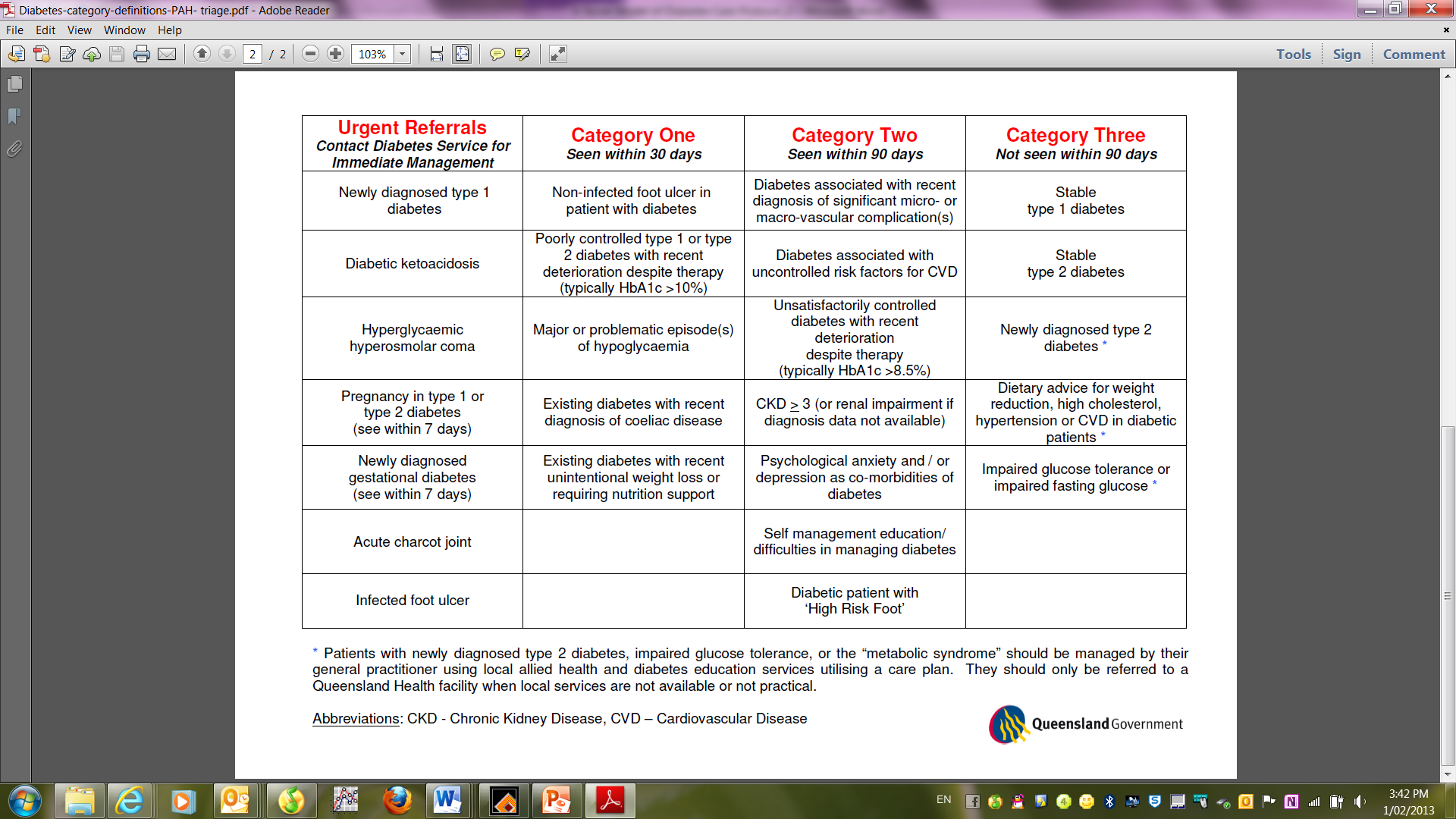

Supplement: Additional file 1: — Triage categories for diabetes services referrals. [file 1745-6215-14-382-S1.doc]
